# A Case Report of Sick Sinus Syndrome as an Initial Presentation of Primary Amyloidosis

**DOI:** 10.7759/cureus.13922

**Published:** 2021-03-16

**Authors:** Basel Abdelazeem, Bilal Malik, Nischit Baral, Rudin Gjeka, Arvind Kunadi

**Affiliations:** 1 Internal Medicine, McLaren Health Care, Flint/Michigan State University, Michigan, USA; 2 Cardiology, McLaren Health Care, Flint/Michigan State University, Michigan, USA; 3 Internal Medicine/Nephrology, McLaren Health Care, Flint/Michigan State University, Michigan, USA

**Keywords:** amyloidosis, sick sinus syndrome, heart failure, heart block, restrictive cardiomyopathy, case report

## Abstract

Primary light chain amyloidosis (AL amyloidosis) rarely presents as sick sinus syndrome (SSS), and only a few cases have been reported in the literature. A higher index of suspicion is needed to diagnose AL amyloidosis in patients presenting with SSS. Recognizing the electrocardiography (ECG) and transthoracic echocardiogram (TTE) findings for amyloidosis are crucial for early recognition, proper management, and to improve the patients' quality of life.

A 79-year-old female initially presented with dyspnea and was diagnosed with SSS that required a pacemaker insertion. Ten days later, the patient had complained of dysphagia and difficulty swallowing. She underwent an esophagogastroduodenoscopy (EGD) to investigate further, and it revealed esophageal and duodenal ulcers, and biopsy was positive for amyloidosis. The patient was worked up for amyloidosis, including bone marrow biopsy, renal biopsy, frees light chains, and serum electrophoresis, which all confirmed the diagnosis of primary amyloidosis. Unfortunately, due to the terminal nature of her condition, the patient was discharged with comfort measures to hospice care.

## Introduction

Primary light chain amyloidosis (AL amyloidosis) is a systemic disorder caused by the deposition of proteinaceous material called amyloid in multiple organs' extracellular space. The cardiovascular presentation includes heart failure, myocardial ischemia, and conductive system pathology. There are numerous etiologies of conduction system abnormalities, of which cardiac amyloidosis accounts for 2.3% [1]. Initial diagnosis may be clinical, with the presentation consisting of conduction system abnormalities, cardiac biomarkers, electrocardiography (ECG), and transthoracic echocardiogram (TTE) findings in patients with proven systemic amyloidosis. A definitive diagnosis requires an endomyocardial biopsy. To the best of our knowledge, only a few cases reported sick sinus syndrome (SSS) as an initial presentation of AL amyloidosis. Herein, we report such a case and highlight the importance of early diagnosis by appropriately identifying ECG and TTE features of AL amyloidosis.

## Case presentation

A 79-year-old African American female presented to the emergency room for shortness of breath with exertion and grade 2+ bilateral lower limb edema. The patient denied dizziness, palpitation, fever, chills, productive cough, abdominal pain, nausea, vomiting, hematemesis, melena, and hematochezia.

**Figure 1:**
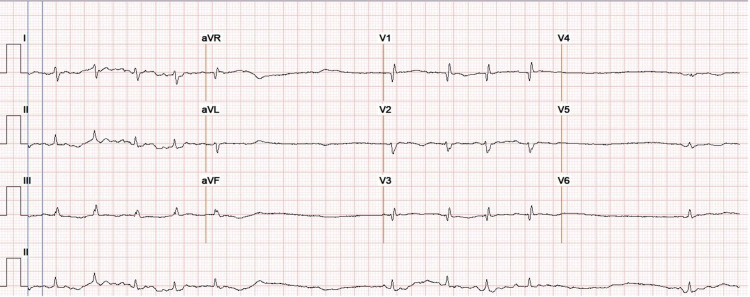
Electrocardiography revealed sick sinus syndrome with sinus bradycardia, pauses up to three seconds, and atrial fibrillation with a rate of 130 bpm.

On examination, the patient's blood pressure was 94/66 mmHg, the temperature was 97.6 °F, the heart rate was irregular with a rate of 78 beats per minute, respiratory rate was 20 breaths per minute, and she was saturating 100% on room air. Sinus pauses were noted, up to 10 seconds in length, on the telemetry monitor. ECG and telemetry monitoring demonstrated SSS with sinus bradycardia, pauses up to three seconds, and atrial fibrillation (AF) with a rate of 130 beats per minute (bpm; Figure 1). Her other medical history included paroxysmal atrial tachycardia, lower extremity venous insufficiency, diastolic heart failure (ejection fraction of 70-75%), chronic obstructive pulmonary disease, and gastroesophageal reflux disease, and no other medical history reported. The patient's home medications included fluticasone propionate/salmeterol inhaler, albuterol sulfate inhaler, torsemide, metolazone, potassium chloride, vitamin D3, pantoprazole, sotalol, and no other heart rate modulating medication reported. Laboratory workup revealed a potassium of 4.5 (3.5-5.1 mM/L), magnesium of 2.3 (1.7-2.7 mg/dL), thyroid-stimulating hormone of 1.54 (0.34-5.60 µIU/mL), and troponin of 0.1 (0.0-0.5 ng/mL). The patient underwent urgent pacemaker placement to prevent further sinus pauses.

The patient was also complaining of dysphagia and difficulty swallowing, for which an esophagogastroduodenoscopy (EGD) was performed. The procedure identified both esophageal and duodenal ulcers. The biopsy was performed during the EGD and sent for histopathological review. The esophagus contained small columnar pieces of mucosa and collections of eosinophilic, amorphous material demonstrated on congo red staining. The congo red stain contained one fragment with blood vessels that exhibited mural deposition of amyloid. In the duodenal biopsy, the lamina propria also had amyloid on congo red staining. Videofluoroscopic swallowing examination revealed no tracheal aspiration and no motility disorder. Eventually, the patient required percutaneous endoscopic gastrostomy tube placement for feeding secondary to dysphagia. TTE findings were consistent with restrictive cardiomyopathy, raising the possibility of cardiomyopathy secondary to amyloidosis and SSS as the initial presenting feature in our patient.

The patient had also developed a bifascicular block, which was potentially consistent with an infiltrative amyloid disease. The patient had a Kappa free light chain drawn, and this was markedly elevated at 146 mg/dL (reference range 0.3-1.9 mg/dL), Lambda 1.97 (0.5-2.6 mg/dL), with a Kappa/Lambda ratio of 74.1. We performed electrophoresis to investigate further and found an elevated gamma globulin of 1.75 g/dL (reference range 0.70-1.50 mg/dL).

**Figure 2:**
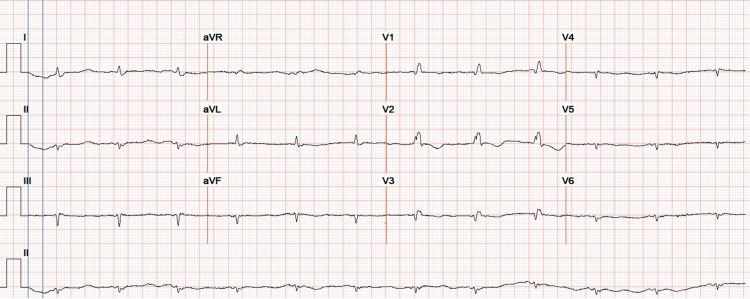
Electrocardiography revealed generalized low voltage QRS with poor R wave progression consistent with amyloidosis.

Two months later the patient presented with persistent, progressive shortness of breath on minimal exertion and persistent lower limb edema. Laboratory work-up was significant for brain natriuretic peptide (BNP) of 838 (2-100 pg/mL), BUN of 37, creatinine of 1.85, white blood cell count of 10.3 (4.5-11.0 × 10^3^/µL), hemoglobin of 10.7 (12.0-15.7 g/dl), and platelet count of 178 (140-440 × 10^3^/µL). ECG was obtained and demonstrated an atrial paced rhythm, bifascicular block, left anterior fascicular block, right bundle branch block, and a pronounced low voltage pattern throughout all leads (Figure 2). She had three negative troponins, and her pacemaker was found to be functioning appropriately. The patient did not have any compelling cardiac symptoms aside from the dyspnea on exertion.

**Figure 3:**
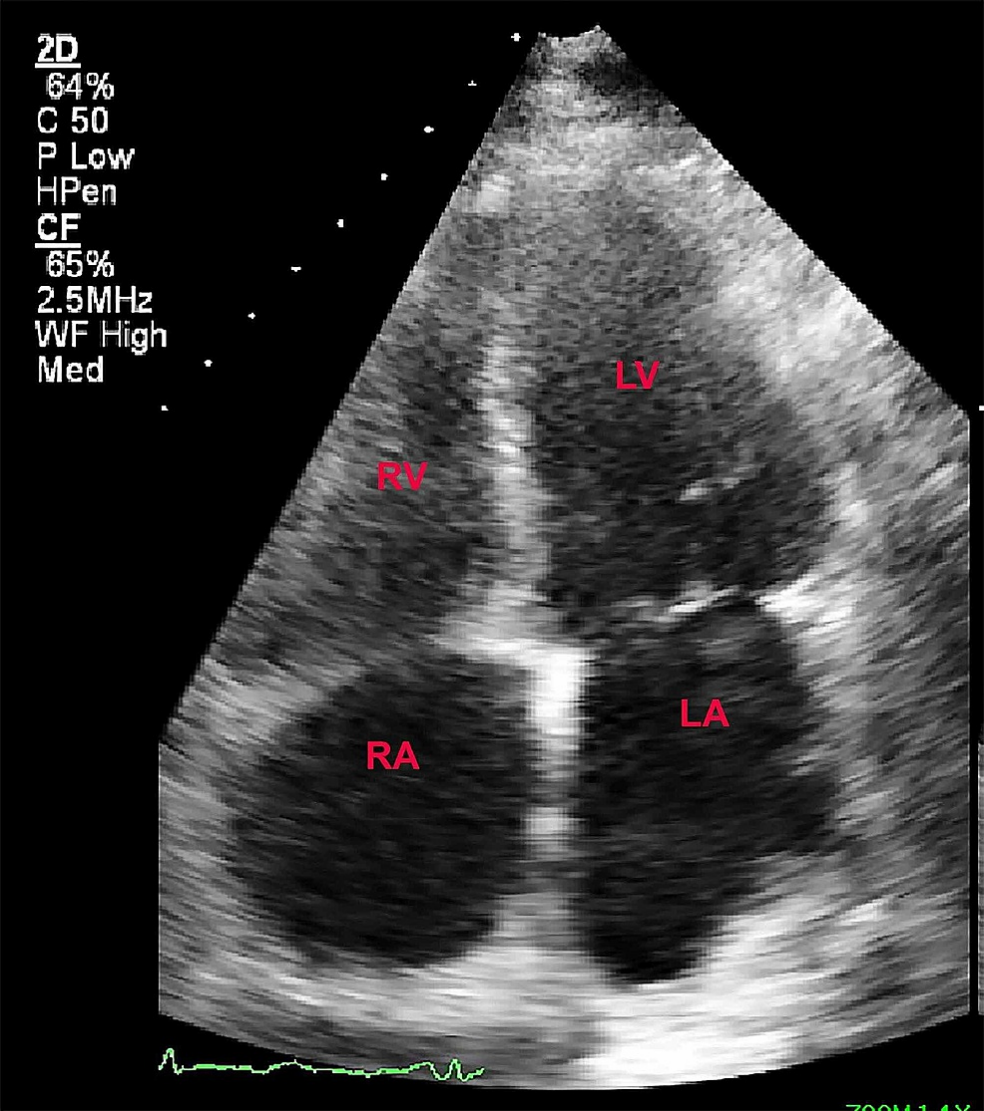
Transthoracic echocardiogram revealed biatrial enlargement.

TTE demonstrated an ejection fraction of 60%, a small pericardial effusion, an increased left ventricular wall thickness (left ventricular posterior wall diastolic thickness 1.4 cm (0.6-1.0/0.6-0.9 cm), right ventricular systolic pressure (RVSP) 56 mmHg, and mild to moderate biatrial enlargement (Figure 3).

A renal biopsy and bone marrow biopsy were performed to confirm the diagnosis of amyloidosis. The results of these biopsies were consistent with our hypothesis that the patient had AL amyloid. She was intermittently treated with Dexamethasone and Bortezomib but ultimately evaluated by palliative care for comfort measures due to the prognosis of her condition.

## Discussion

Cardiac amyloidosis classically presents with clinical features of restrictive cardiomyopathy, right ventricular failure, arrhythmias, and possible angina due to deposition of the amyloid protein in the heart's microvasculature [2]. Conduction system abnormalities are a potential complication of amyloidosis due to amyloid deposits' infiltration of the conduction system [1]. The physical examination may include periorbital bruising, macroglossia, and carpal tunnel syndrome [2]. Initial laboratory workup for amyloidosis includes Troponin T and N-terminal pro-B-type natriuretic peptide (NT-proBNP), which may be elevated. ECG findings in cardiac amyloidosis are summarized in (Table 1).

**Table 1:** Electrocardiography findings in cardiac amyloidosis.

ECG findings in cardiac amyloidosis
Low-voltage QRS (<5 mm in height in limb leads) with poor R- wave progression in the chest leads (up to 50%).
First-degree atrioventricular block (21%).
Nonspecific intraventricular conduction delay (16%).
Second- or third-degree atrioventricular block (3%).
Atrial fibrillation/flutter (20%).
Ventricular tachycardia (5%).

TTE features that support a diagnosis of cardiac amyloidosis include increased left ventricular wall thickening, low ventricular volumes (in both systolic and diastolic phases), biatrial enlargement, mildly thickened valves, and trivial pericardial effusion. Endomyocardial biopsy is the gold standard test for diagnosis and is required if isolated cardiac amyloidosis is suspected in AL amyloidosis [3]. In transthyretin amyloidosis (ATTR), the absence of monoclonal proteins and grades 2-3 uptake on nuclear imaging has a specificity of 100%, a positive predictive value of 100%; thus, no biopsy is required. In patients with proven systemic amyloidosis with multi-organ involvement and the clinical presentation of heart failure, cardiac amyloidosis can be diagnosed using ECG, TTE, and cardiac biomarkers. Our patient initially presented with dyspnea secondary to SSS. The ECG demonstrated SSS (Tachy-Brady syndrome), with the follow-up ECG exhibiting a paced sinus rhythm with low-voltage QRS complexes and poor R wave progression. The TTE revealed features consistent with restrictive cardiomyopathy. The follow-up TTE was remarkable for concentric hypertrophy of the left ventricular wall, mild biatrial enlargement, and a small pericardial effusion. Specimens from both the EGD biopsies and renal biopsies were consistent with a diagnosis of amyloidosis. In advanced stages of amyloidosis, significant diastolic dysfunction is frequently present. Our patient's TTE revealed significant diastolic dysfunction with a lateral velocity of 3.5 cm/sec and medial velocity of 2.8 cm/sec. The left ventricular wall thickness and RVSP were increased. Apical sparing with global ventricular strain is typical in cardiac amyloidosis, but the ventricular straining part of the TTE was not done in our patient. Due to the multi-organ involvement and characteristic findings on the above studies, the endomyocardial biopsy was not indicated to confirm the diagnosis of amyloidosis in our patient.

Another potential complication of cardiac amyloidosis includes atrial or intracardiac thrombus, with a prevalence of up to 30% in cardiac amyloidosis patients. Transesophageal echocardiography (TEE) is the study of choice for the evaluation of atrial or intracardiac thrombus [4]. Treatment strategies consist of supportive treatment for heart failure and rhythm disturbances, including cardiac devices (e.g., implantable cardioverter defibrillator, left ventricular assist devices, and permanent pacemaker implantation) [2]. In cardiac amyloidosis patients with heart failure and preserved ejection fraction, traditional neurohumoral therapies have not been proven effective and, in some cases, were associated with worse outcomes [5].

Chemotherapy, stem cell transplantation, and cardiac transplant may also be considered in selected patients [6]. Early recognition and intervention are essential factors in determining prognosis and improving quality of life. Unfortunately, the overall prognosis in cardiac amyloidosis is very poor. In tafamidis treatment for patients with transthyretin amyloid cardiomyopathy clinical trial (ATTR-ACT), there was a 57.1% survival rate in the placebo versus a 70.5% survival rate in the tafamidis group at 30 months [6].

Our patient's atypical presentation, characterized by SSS, underscores the need to consider cardiac amyloidosis in patients presenting with SSS with TTE findings characteristic of restrictive cardiomyopathy. To the best of our knowledge, very few cases of SSS as the initial presentation of AL amyloidosis have been documented and reported; age, gender, and initial ECG features from available observational studies with similar presentations have been summarized in (Table 2).

**Table 2:** Age, gender, and initial electrocardiography features on presentation in patients with sick sinus syndrome and amyloidosis (via multiple case reports). AV: atrioventricular, AF: atrial fibrillation, SA: sinoatrial, LBBB: left bundle branch block, RBBB: right bundle branch block.

Author	Age	Gender	Associated EKG features on presentation
Gilotra et al. [3]	66	Female	Low-voltages, pseudo infarction pattern, QS waves in anteroseptal leads, prolonged QTc 550 ms.
Narumi et al. [7]	63	Male	Junctional rhythm with HR 45 bpm.
Li et al. [8]	66	Female	Low-voltage, bradyarrhythmia with junctional escape beats, complete RBBB, prolonged PR interval.
Olofsson et al. [9]	48	Male	Sinus bradycardia, SA block, AV junctional escape rhythm (intermittent), incomplete RBBB, AV block I.
	42	Female	SA block, AV junctional escape rhythm (intermittent), AV block I.
	73	Male	AF with bradycardia (30-40 bpm), incomplete RBBB, left anterior fascicular block.
	65	Male	SA block, paroxysmal atrial tachycardia, LBBB, AV block I.
	70	Male	Sinus bradycardia, intermittent AV junctional escape rhythm, LBBB, AV block I.
Pattanshettyn et al. [10]	76	Male	AF with a ventricular rate of 70-80 bpm, RBBB, normal voltage complexes, irregularly irregular rhythm.

## Conclusions

AL amyloidosis is a rare disease that can be present with SSS. Screening for cardiac involvement should be considered in all patients with amyloidosis. We presented a case of a 79-year-old African American female who presented with SSS as the initial presentation, complicated by dysphagia and restrictive cardiomyopathy, and was diagnosed with AL amyloidosis. This case highlights the importance of early recognition of AL amyloidosis by ECG and TTE to improve patients' quality of life.
